# Screening the Biosphere: The Fungicolous Fungus *Trichoderma phellinicola*, a Prolific Source of Hypophellins, New 17-, 18-, 19-, and 20-Residue Peptaibiotics1)

**DOI:** 10.1002/cbdv.201200339

**Published:** 2013-05-17

**Authors:** Christian René Röhrich, Anita Iversen, Walter Michael Jaklitsch, Hermann Voglmayr, Andreas Vilcinskas, Kristian Fog Nielsen, Ulf Thrane, Hans von Döhren, Hans Brückner, Thomas Degenkolb

**Affiliations:** a) Fraunhofer Institute for Molecular Biology and Applied Ecology (IME), Bioresources Project GroupWinchesterstrasse 2, D-35394 Giessen (*C. R. R.:* phone: +49-641-99-37617, e-mail: christian.roehrich@ime.fraunhofer.de; A. V.: phone: +49-641-99-39500, fax: +49-641-4808-581, e-mail: andreas.vilcinskas@ime.fraunhofer.de); b) Department of Systems Biology, Center for Microbial Biotechnology, Technical University of Denmark (DTU)Søltofts Plads, Building 221, DK-2800 Kgs. Lyngby (*A. I.:* phone: +45-45252725, e-mail: aive@bio.dtu.dk; K. F. N.: phone: +45-45252602, fax: +45-45884922, e-mail: kfn@bio.dtu.dk; *U. T*.: phone: +45-45252630, fax: 45-45884148, e-mail: ut@bio.dtu.dk); c) Department of Systematic and Evolutionary Botany, Faculty Centre of Biodiversity, University of ViennaRennweg 14, A-1030 Vienna (*W. M. J.:* phone: +43-1-4277-54055, e-mail: walter.jaklitsch@univie.ac.at; H. V.: phone: +43-4277-54050, e-mail: hermann.voglmayr@univie.ac.at); d) Interdisciplinary Research Centre for BioSystems, Land Use and Nutrition (IFZ), Department of Applied Entomology, Institute of Phytopathology and Applied Zoology (IPAZ), University of Giessen (JLU)Heinrich-Buff-Ring 26–32, D-35392 Gießen (phone: +49-641-99-37601; e-mail: thomas.degenkolb@ernaehrung.uni-giessen.de); e) Biochemistry and Molecular Biology OE 2, Institute of Chemistry, Technical University of BerlinFranklinstraße 29, D-10587 Berlin (phone: +49-30-314-22697; fax: +49-30-314-24783; e-mail: doehren@chem.tu-berlin.de); f) Interdisciplinary Research Centre for BioSystems, Land Use and Nutrition (IFZ), Department of Food Sciences, Institute of Nutritional Science, University of GiessenHeinrich-Buff-Ring 26–32, D-35392 Gießen (phone: +49-711-349919; e-mail: hans.brueckner@ernaehrung.uni-giessen.de)

## Abstract

To investigate the significance of antibiotics for the producing organism(s) in the natural habitat, we screened a specimen of the fungicolous fungus *Trichoderma phellinicola* (syn. *Hypocrea phellinicola*) growing on its natural host *Phellinus ferruginosus*. Results revealed that a particular group of non-ribosomal antibiotic polypeptides, peptaibiotics, which contain the non-proteinogenic marker amino acid, *α*-aminoisobutyric acid, was biosynthesized in the natural habitat by the fungicolous producer and, consequently, released into the host. By means of liquid chromatography coupled to electrospray high-resolution time-of-flight mass spectrometry, we detected ten 20-residue peptaibols in the specimen. Sequences of peptaibiotics found *in vivo* were independently confirmed by analyzing the peptaibiome of an agar plate culture of *T. phellinicola* CBS 119283 (*ex*-*type*) grown under laboratory conditions. Notably, this strain could be identified as a potent producer of 39 new 17-, 18-, and 19-residue peptaibiotics, which display the same building scheme as the 20-residue peptaibols found in the specimen. Two of the 19-residue peptaibols are tentatively assigned to carry tyrosinol, a novel C-terminal residue, as deduced from high-resolution tandem mass-spectrometry data. For the new peptaibiotics produced by *T. phellinicola*, the name ‘hypophellin(s)’, based on the teleomorph name, is introduced.

## 1. Introduction

### 1.1. Fungi as a Prolific Source of Bioactive Natural Products

The current estimate of the total number of fungal species ranges between 1.0 and 1.5 million [1], whereas the number of those validly described should now exceed only 98,000 [2]. Of the 33,500 bioactive microbial metabolites known to date, the fungal kingdom contributes *ca*. 15,600. Approximately 10,000 of them were shown to display anti-infective, antitumor, and/or antiviral activities. Microbial-derived drugs on the market comprise *ca*. 400–500 active pharmaceutical agents [3], including therapeutically relevant antibiotics of fungal origin such as β-lactams, fusidic acid, and griseofulvin, as well as the two immunosuppressants mycophenolic acid and cyclosporin A [4].

Given that less than 1% of microorganisms visible under the microscope have been cultivated under laboratory conditions so far, microbial diversity provides an enormous, yet underestimated potential for future drug discovery [5] and in the search for new agricultural antibiotics [6].

### 1.2. The Potential of *Trichoderma* Species as Biological Control Agents *(BCAs)*

Species of the ubiquitous fungal genus *Trichoderma* and its *Hypocrea* teleomorphs have attracted considerable interest in the past two decades because of the pivotal role of their secondary metabolites in the antagonistic activities of biocontrol species [7–9]. Most of them occur as opportunistic, plant (endo)symbionts [10], some of which exhibit pronounced antimicrobial activity towards economically important plant pathogens. Recent examples include:

- the hyperparasite *Trichoderma stromaticum* (syn. *Hypocrea stromatica)*, the active agent of ‘*Tricovab’* a commercial formulation against *Crinipellis* (syn. *Moniliophthora) perniciosa*, the Witches’ broom pathogen of cocoa *(Theobroma cacao)* [11] [12];
- *T paucisporum* and *T theobromicola*, displaying *in* vitro-activities against frosty pod rot of cocoa, *Moniliophthora roreri* [13];
- *T martiale*, which, in small-scale *in situ* field trials, proved highly effective against black pod rot of cocoa caused by *Phytophthora palmivora* [14].

The mode of action of phytoprotective *Trichoderma* species is considered rather complex. Depending on the species or even strains investigated, the following mechanisms may contribute to the antagonistic potential towards plant pathogenic fungi:

*i)* Competition for nutrients and/or space, *ii)* growth promotion of plants, especially colonization of roots, resulting in improved root and plant growth, ii*i)* induction of localized and systemic resistance responses in plants, *iv)* mycoparasitism, v) increase of uptake and concentration of nutrients by the plant, including the production of siderophores, and *vi)* production of volatile and non-volatile antibiotics [10].

### 1.3. Peptaibiotics – Non-Ribosomally Biosynthesized Fungal Peptide Antibiotics Containing α,α-Dialkyl-α-amino Acids

**Fig. 1:**
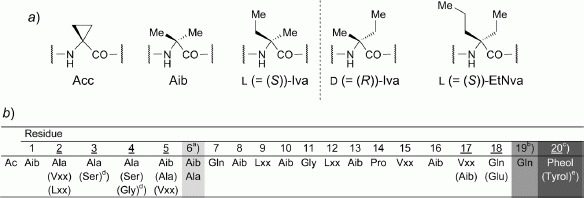
a) *Structures and configurations of a,a-dialkylamino acids found in peptaibiotics*. b) *Building scheme of subfamily-1* (SF1) *peptaibiotics, produced by* Hypocrea phellinicola. Variable positions are underlined. Minor sequence variations are parenthesized. Deletions of certain amino acid positions are highlighted in different shades: C-terminal deletions are highlighted in dark, deletions of Gln in medium, and deletions of [Aib/Ala]^6^ in light gray. **a**) Deleted in 17-, 18-, and 19-residues hypophellins. **b**) Deleted in the 17-residue sequence **29**. **c**) Deleted in 18-residue sequences **11, 12**, and **28**, and in the 17-residue sequence **29**. **d**) Detected with DTU *maXis* gradient only. **e**) Detected with JLU *micrOTOF-Q II* gradient only.

During the past two decades, peptaibiotics have regained particular interest because of their unique bioactivities, resulting from their amphipathicity and helical conformations [15]. These are attributed to the presence of high proportions of peptide-bound α-aminoisobutyric acid (Aib), frequently accompanied by d- and/or l-isovaline (Iva) [16], and, in a few sequences, l-α-ethylnorvaline (EtNva), or 1-aminocyclopropane-1-carboxylic acid (Acc) [17]. The presence of these α,α-dialkyl-α-amino acids (Fig. 1,*a*) has been confirmed in acidic hydrolysates of more than 30 genera of fungi [18].

Peptaibiotics are defined as non-ribosomally biosynthesised, linear or cyclic polypeptide antibiotics of exclusively fungal origin which *i*) have a molecular weight between 500 and 2,200 Da, thus containing 4–21 residues; *ii*) show a high content of the marker Aib, as well as further α,α-dialkylamino acids; *iii*) are characterized by the presence of other non-proteinogenic amino acids and/or lipoamino acids; *iv*) possess an acylated N-terminus, and *v*) in the case of linear peptides, have a C-terminal residue that, in most of them, consists of a free or *O*-acetylated, amide-bonded β-amino alcohol. The C-terminus might also be an amine, amide, sugar alcohol, 2,5-diketopiperazine, a heterocyclic residue, or an amino acid with free carboxy terminus. The majority of Aib-containing peptides carry a C-terminal residue representing a β-amino alcohol. Only this group is referred to as *peptaibols sensu stricto*, whereas for the others the comprehensive name peptaibiotics is used [17].

### 1.4. Detection of Peptaibiotics in *T. phellinicola* Growing on Its Natural Host

The genus Trichoderma, which currently consists of ca. 200 validly described species the number of which increases continually [19–28], is generally recognized as the most prolific source of peptaibiotics [17]. However, reports on the detection of peptaibiotics in samples collected in the natural habitat of the producer(s) are rare. Most of the *ca*. 1,000 individual sequences of peptaibiotics known to date have been sequenced in extracts of fungal cultures grown under artificial laboratory conditions.

The first example of peptaibiotics isolated from natural specimens were hypelcins A and B obtained from *ca*. 2 kg of dried, crushed stromata of *Hypocrea peltata* [29–31]. In 1997 and 1999, three reports were published on the isolation of peptaibiotics from fruiting bodies of *Scleroderma texense, Tylopilus neofelleus*, and *Boletus* sp., respectively; all being members of the Boletales [32–34]. However, in 2002, *Kiet et al*. [35] isolated chrysospermins A–D from the Vietnamese species *Xerocomus langbianensis* (Boletaceae, Boletales) and attributed the detection of these four 19-residue peptaibols [36] to an unrecognized infection of *X. langbianensis* with *Sepedonium* sp. This phenomenon was later commented on by *Degenkolb et al*. [37] [38]. Finally, *Neuhof et al*. [39] corroborated the assumption of *Kiet et al*. [35] by analyzing four fruiting bodies of members of the order Boletales infected by *Sepedonium chrysospermum* and *S. microspermum*, respectively. Notably, all samples were screened positive for peptaibiotics of the chrysospermin type. In 2006, *Lehr et al*. [40] demonstrated that 16-residue peptaibols, the antiamoebins, were solely responsible for antibiosis in herbivore dung naturally colonized by or artificially inoculated with *Stilbella fimetaria* (syn. *S. erythrocephala*).

### 1.5. Bioactivities of Peptaibiotics from *Trichoderma*

Peptaibiotics are thus assumed to play a key role in the infection process of a host by a fungicolous species because of their unique ability of forming voltage-gated ion channels. This phenomenon is best described by the dipole flip-flop gating model in planar lipid bilayers [41]. Their well-documented membrane activity, however, may also account for other striking bioactivities, such as neurolepsy [42], inhibition of amyloid β-peptide formation [43], inhibition of HIV-1 integrase [44], suppression of tumor cells, targeted calcium-mediated apoptosis, and autophagy in human hepatocellular carcinoma cells [45], as well as induction of defence responses and systemic resistance in tobacco against tobacco mosaic virus [46] and programmed cell death in fungal plant pathogens [47].

### 1.6. Choice of the Model Organism

*Trichoderma phellinicola*, a recently described polyporicolous species, which specifically occurs on effused basidiomes of *Phellinus* spp., was chosen as a model organism. Specimens of *H. phellinicola* have so far been recorded from Austria, Denmark, Germany [20], and the Czech Republic (see *Exper. Part)*. This species is possibly specific for *Phellinus ferruginosus* [20].

To confirm the above hypothesis of peptaibiotic production under *in vivo* conditions, a specimen of *Trichoderma phellinicola* growing on its host *Phellinus ferruginosus*, was screened for peptaibiotics. For comparison, the *ex-type* culture of *T. phellinicola*, CBS 119283 (= C.P.K. 2137), was investigated. Both morphs were analyzed using a peptaibiomics approach as described in [48–50].

## 2. Results

### 2.1. General Considerations

All 17-, 18-, 19-, and 20-residue sequences discussed below were obtained from *Trichoderma phellinicola* [20]. The name ‘*hypophellins*’ (HPHs), which covers the entirety of long-chain peptaibiotics (>17 residues) produced by this species, is proposed. We base this name on the teleomorph name *Hypocrea phellinicola*, which used to be the valid name of the holomorph in dual nomenclature [20]. The introduction of a new name for peptaibiotics from a phylogenetically well-defined species is more favorable than earlier names for many of the 19- and 20-residue peptaibiotics mentioned below, *viz*. suzukacillins, trichocel-lins, trichokonins, and longibrachins, which were produced by phylogenetically undefined *Trichoderma* species with thus highly questionable names. The latter issue is further complicated by the fact that many of the peptaibiotic-producing *Trichoderma* strains reported in the literature have never been deposited in a public culture collection, or deposition was terminated [51].

Hypophellins are numbered consecutively with Arabic numbers as follows: *i)* sequences produced by the specimen; *ii)* sequences produced by the culture CBS 119283 grown and analyzed at JLU; *iii)* sequences produced by the culture CBS 119283 grown and analysed at DTU.

### 2.2. Peptaibiotic Pattern of the Teleomorph

Notably, the teleomorph of *Trichoderma phellinicola* proved to be a prolific source of ten 20-residue peptaibols, compounds **1**-**10**, displaying the characteristic building scheme of subfamily 1 (SF1), one of the nine ‘peptaibol subfamilies’ (Fig. 1,*b*, and *Tables 1* and *2*), as introduced by *Chugh* and *Wallace* [52]4).

**Table 1:** Sequences of 20-Residue Peptaibiotics Detected in the Specimen of *Hypocrea phellinicola* (micrOTOF-Q II screening)

No.	*t*_R_ [min]	[*M*+*H*]^+^		Residue										
				
				1	2a)	3	4	5	6	7	8	9	10	11
**1**	37.8–38.1	1937.1209	Ac	Aib	Ala	Aib	Ala	Aib	Ala	Gln	Aib	Vxx	Aib	Gly
**2**	37.8–38.1	1938.1068	Ac	Aib	Ala	Aib	Ala	Aib	Ala	Gln	Aib	Vxx	Aib	Gly
**3**	39.1–39.3	1951.1358	Ac	Aib	Ala	Aib	Ala	Aib	Ala	Gln	Aib	Vxx	Aib	Gly
**4**	39.8–40.0	1952.1192	Ac	Aib	Ala	Aib	Ala	Aib	Ala	Gln	Aib	Vxx	Aib	Gly
**5**	40.2–40.4	1951.1416	Ac	Aib	Ala	Aib	Ala	Aib	Aib	Gln	Aib	Vxx	Aib	Gly
**6**	41.0–41.2	1952.1258	Ac	Aib	Ala	Aib	Ala	Aib	Aib	Gln	Aib	Vxx	Aib	Gly
**7**	41.3–41.7	1965.1615	Ac	Aib	Ala	Aib	Ala	Aib	Aib	Gln	Aib	Vxx	Aib	Gly
**8**	42.3–42.5	1966.1354	Ac	Aib	Ala	Aib	Ala	Aib	Aib	Gln	Aib	Vxx	Aib	Gly
**9**	43.0–43.2	1979.1718	Ac	Aib	Aib	Aib	Ala	Aib	Aib	Gln	Aib	Vxx	Aib	Gly
**10**	44.0–44.3	1980.1636	Ac	Aib	Aib	Aib	Ala	Aib	Aib	Gln	Aib	Vxx	Aib	Gly

									Compound identical or positionally isomeric with	Ref.
		
12	13	14	15	16	17	18	19	20		
Lxx	Aib	Pro	Vxx	Aib	Aib	Gln	Gln	Pheol	Longibrachin A I	[53]
									Trichoaureocin 3	[54]
									Trichokonin VI (= gliodeliquescin A)	[55] [56]
									Trichobrachins II-5, II-6	[57]
									Trichobrachin IIb A	[58] [59]
Lxx	Aib	Pro	Vxx	Aib	Aib	Glu	Gln	Pheol	Longibrachin B II	[53]
Lxx	Aib	Pro	Vxx	Aib	Vxx	Gln	Gln	Pheol	Trichokonin VII	[55]
									Trichoaureocin 4	[54]
									Suzukacillin A-10a	[60]
									Trichobrachins II-7, II-8, II-9	[57]
									Trichobrachin IIb B	[58] [59]
Lxx	Aib	Pro	Vxx	Aib	Vxx	Glu	Gln	Pheol	Longibrachin B III	[53]
Lxx	Aib	Pro	Vxx	Aib	Aib	Gln	Gln	Pheol	Trichokonin VIII (= trichosporin B-IVc)	[55] [61]
									Trichoaureocin V	[54]
									Trichobrachin IIb C	[58] [59]
Lxx	Aib	Pro	Vxx	Aib	Aib	Glu	Gln	Pheol	**New**	
Lxx	Aib	Pro	Vxx	Aib	Vxx	Gln	Gln	Pheol	Longibrachin A IV	[53]
									Trichoaureocin VI	[54]
									Trichobrachin IIb D	[58] [59]
Lxx	Aib	Pro	Vxx	Aib	Vxx	Glu	Gln	Pheol	**New** (longibrachin IV: [Gln]^18^→[Glu]^18^)	
Lxx	Aib	Pro	Vxx	Aib	Vxx	Gln	Gln	Pheol	**New** (homolog of **7**)	
Lxx	Aib	Pro	Vxx	Aib	Vxx	Glu	Gln	Pheol	**New** (homolog of **8**)	

a) Variable residues are underlined in the table header. Minor sequence variants are underlined in the sequences. This applies to *Tables 1, 3*, and *5*.

**Table 2:** Diagnostic Fragment Ions of 20-Residue Peptaibiotics Detected in the Specimen of *Hypocrea phellinicola* (micrOTOF-Q II screening)

Diagnostic fragment ions	Peaks [*m/z*]a)									
	
	1	2	3	4	5	6	7	8	9	10

*t*_R_ [min]	37.8–38.1	38.6–38.7	39.1–39.3	39.8–40.0	40.2–40.4	41.0–41.2	41.3–41.7	42.3–42.5	43.0–43.2	44.0–44.3
[*M* + Na]^+^	1959.1047	1960.0872	1962.1376	n.d.	1973.1212	1974.1064	1987.1372	1988.1245	2001.1535	2002.1445
[*M* + H]^+^	1937.1209	1938.1036	1951.1358	1952.1192	1951.1416	1952.1258	1965.1615	1966.1354	1979.1718	1980.1636
*a*_1_	100.0808	100.0808	100.0808	100.0806	100.0809	100.0805	100.0808	100.0807	n.d.	n.d.
*a*_2_	171.1181	171.1181	171.1197	171.1195	171.1188	171.1185	171.1191	171.1200	185.1315	185.1311
*a*_3_	256.1657	256.1657	256.1662	256.1663	256.1666	256.1665	256.1669	256.1671	270.1811	270.1808
*a*_4_	327.2121	327.2121	327.2155	327.2142	327.1992	327.2097	327.2102	327.2116	341.2238	341.2180
*a*_5_	n.d.	n.d.	412.2739	412.2739	412.2572	412.2720	412.2776	412.2766	n.d.	n.d.
*b*_1_	128.0758	128.0758	128.0762	128.0757	128.0763	128.0758	128.0765	128.0760	128.0758	128.0748
*b*_2_	199.1102	199.1102	199.1109	199.1107	199.1111	199.1111	199.1113	199.1115	213.1251	213.1251
*b*_3_	284.1604	284.1604	284.1615	284.1614	284.1618	284.1615	284.1623	284.1625	298.1845	298.1802
*b*_4_	355.1982	355.1982	355.1973	355.1972	355.1981	355.1976	355.1986	355.1988	369.2109	369.2144
*b*_5_	440.2479	440.2479	440.2494	440.2492	440.2502	440.1497	440.2508	440.2510	454.2669	454.2699
*b*_6_	511.2839	511.2839	511.2850	511.2852	525.3019	525.3015	525.3023	525.3026	539.3231	539.3231
*b*_7_	639.3431	639.3431	639.3455	639.3451	653.3661	653.3626	653.3690	653.3681	667.3870	667.3881
*b*_8_	724.3937	724.3937	724.3961	724.3957	738.4118	738.4109	738.4130	738.4132	752.4381	752.4367
*b*_9_	823.4750	823.4590	823.4611	823.4601	837.4777	837.4772	837.4790	837.4795	851.5058	851.5067
*b*_10_	908.5298	908.5095	908.5131	908.5129	922.5302	922.5290	922.5314	922.5317	936.5594	936.5602
*b*_11_	965.5490	965.5311	965.5470	965.5316	979.5501	979.5476	979.5508	979.5506	993.5822	993.5819
*b*_12_	1078.6366	1078.6151	1078.6340	1078.6138	1092.6478	1092.6289	1092.6325	1092.6326	1106.6642	1106.6710
*b*_13_	1163.6824	1163.6642	1163.6810	1163.6662	1177.6994	1177.6816	1177.6853	1177.6859	1191.7215	1191.7196
*y*_7_	774.4598	775.4614	788.4742	789.4595	774.4586	775.4436	788.4750	789.4596	788.4750	789.4596
*y*_7_ – H_2_O	756.4445	757.4491	n.d.	771.4507	n.d.	757.4308	n.d.	771.4468	n.d.	771.4468
*y*_7_ – AA (20)	623.3556	624.3581	637.3711	638.3573	623.3563	624.3414	637.3722	638.3576	637.3722	638.3576
*y*_7_ – AA (20-19)	495.2979	496.2999	509.3124	510.2961	495.2955	496.2793	509.3120	510.2962	509.3120	510.2962
*y*_7_ – AA (20-18)	367.2385	367.2350	381.2515	381.2517	367.2364	367.2358	381.2519	381.2514	381.2519	381.2514
*y*_7_ – AA (20-17)	282.1900	282.1831	282.1850	282.1820	282.1853	282.1838	282.1839	282.1839	282.1839	282.1839

a) n.d., Not detected.

One Gln residue is found in position 7, and another one towards or at the C-terminus in position 18, whereas position 19 is either occupied by a third Gln or a Glu residue. A highly conserved Pro residue is located in position 14 of the peptide chain. All sequences have a Gly residue in position 11 and terminate in Pheol. At least seven, at most nine, residues are occupied by Aib. Variable amino acid residues are located in positions 2, 6, 17, and 18 (*Fig. 1*,*b*).

**Fig. 2:**
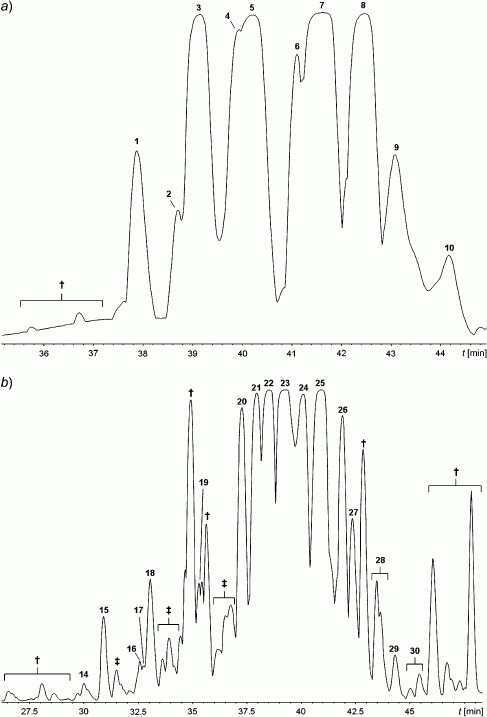

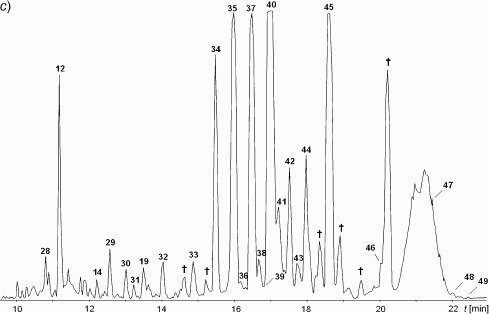
*Base-peak chromatograms* (BPCs) *of* a) *the* H. phellinicola *specimen screened with the* micrOTOF-Q II, b) *the* H. phellinicola *ex-type plate culture screened with the* micrOTOF-Q II, *and* c) *the* H. phellinicola *specimen screened with the* maXis. †, co-eluting peptaibiotics, not sequenced; ‡, non-peptaibiotic metabolite.

Most of the peptaibols sequenced resemble previously described compounds (*Fig. 1*,*b*, *Table 1*, and *Fig. 2,a)* such as longibrachins A and B [53], trichobrachins II [57], trichoaureocins [54], trichokonins [55] [62] [63], and suzukacillins A [60].

### 2.3. Peptaibiotic Pattern of the *Culture*

#### 2.3.1. General Considerations

As observed before [20], ascospores of *T phellinicola* are unstable and die rapidly after collecting. This might have been the reason why no agar culture could be obtained from our specimen. As a substitute, the *ex-type* culture of *T phellinicola* CBS 119283 (= C.P.K. 2137) was provided, and its peptaibiotic pattern was analyzed. Except for the two lipopeptaibols **48** and **49**, the remaining compounds **11**–**47** represent the characteristic building scheme of SF1, resembling the previously described 20-residue peptaibols suzukacillins A, trichosporins B, and trichocellins A [60] [61] [64–67].

#### 2.3.2. *micrOTOF-Q II* Screening

**Fig. 3:**
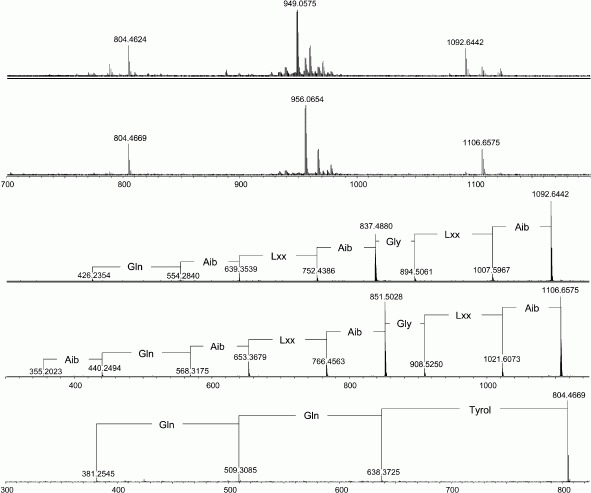
*Sequencing of compounds* **13** *and* **15** *ocontaining a new C-terminal residue with a peak at* m/z *804.46, tentatively assigned as tyrosinol* (Tyrol)

In contrast to the specimen analyzed, the *ex-type* plate culture grown and analyzed at the *Justus Liebig* University of Giessen (JLU) produced two new 18- and fifteen new 19-residue peptaibols, compounds **11–27**, which lacked the [Ala/Aib]^6^ residue of the 20-residue peptaibols found in the specimen *(Tables 3* and *4*, and *Fig. 2,b)*. The two truncated 18-residue sequences, compounds **11** and **12**, terminated in free Gln. Sequences **14** and **16–27** carry a C-terminal Pheol. For compounds **13** and **15**, a C-terminal tyrosinol residue (abbreviated as ‘Tyrol’) was tentatively deduced from HR-ESI-MS/MS data *(Tables 3* and *4, Fig. 3)*.

**Table 3:** Sequences of 18- and 19-Residue Peptaibiotics Detected in the Ex-Type Culture (*CBS 119283*) of *Hypocrea phellinicola* (micrOTOF-Q II screening)

No.	*t*_R_ [min]	[*M* + *H*]^+^		Residue										
				
				1	2	3	4	5	6	7	8	9	10	11
**11**	30.9–31.1	1747.0135	Ac	Aib	Ala	Aib	Ala	Ala	–	Gln	Aib	Lxx	Aib	Gly
**12**	31.8–32.0	1761.0324	Ac	Aib	Ala	Aib	Ala	Aib	–	Gln	Aib	Lxx	Aib	Gly
**13**	32.2–32.6	1896.0995	Ac	Aib	Ala	Ala	Ala	Aib	–	Gln	Aib	Lxx	Aib	Gly
**14**	32.5–32.7	1910.1131	Ac	Aib	Ala	Aib	Ser	Aib	–	Gln	Aib	Lxx	Aib	Gly
**15**	32.8–33.1	1910.1140	Ac	Aib	Ala	Aib	Ala	Aib	–	Gln	Aib	Lxx	Aib	Gly
**16**	35.1–35.3	1896.1035	Ac	Aib	Ala	Ala	Ser	Aib	–	Gln	Aib	Lxx	Aib	Gly
**17**	37.0–37.2	1866.0928	Ac	Aib	Ala	Ala	Ala	Aib	–	Gln	Aib	Lxx	Aib	Gly
**18**	37.7–37.9	1880.1095	Ac	Aib	Ala	Aib	Ala	Aib	–	Gln	Aib	Lxx	Aib	Gly
**19**	38.3–38.4	1880.1136	Ac	Aib	Ala	Ala	Ala	Aib	–	Gln	Aib	Lxx	Aib	Gly
**20**	38.8–39.2	1894.1331	Ac	Aib	Ala	Aib	Ala	Aib	–	Gln	Aib	Lxx	Aib	Gly
**21**	39.8–40.1	1895.1278	Ac	Aib	Ala	Aib	Ala	Aib	–	Gln	Aib	Lxx	Aib	Gly
**22**	40.6–40.9	1908.1474	Ac	Aib	Ala	Aib	Ala	Vxx	–	Gln	Aib	Lxx	Aib	Gly
**23**	41.5–41.6	1909.1391	Ac	Aib	Ala	Aib	Ala	Vxx	–	Gln	Aib	Lxx	Aib	Gly
**24**	42.1–42.3	1922.1601	Ac	Aib	[255]		Ala	Aib	–	Gln	Aib	Lxx	Aib	Gly
**25**	43.4–43.6	1936.1738	Ac	Aib	[269]		Ala	Aib	–	Gln	Aib	Lxx	Aib	Gly
**26**	44.2–44.4	1936.1750	Ac	Aib	Vxx	Aib	Ala	Vxx	–	Gln	Aib	Lxx	Aib	Gly
**27**	45.0–45.6	1950.1894	Ac	Aib	Lxx	Aib	Ala	Vxx	–	Gln	Aib	Lxx	Aib	Gly

									Compound identical or positionally isomeric with	Ref.
		
12	13	14	15	16	17	18	19	20		
Lxx	Aib	Pro	Vxx	Aib	Vxx	Gln	Gln		**New** (trichocellin A-VI – [Aib]^5^ – Pheol)	[67]
Lxx	Aib	Pro	Vxx	Aib	Vxx	Gln	Gln		**New** (trichocellin A-VI – [Ala]^6^ – Pheol)	[67]
Lxx	Aib	Pro	Vxx	Aib	Vxx	Gln	Gln	Tyrol	**New**	
Lxx	Aib	Pro	Vxx	Aib	Vxx	Gln	Gln	Pheol	**New**	
Lxx	Aib	Pro	Vxx	Aib	Vxx	Gln	Gln	Tyrol	**New**	
Lxx	Aib	Pro	Vxx	Aib	Vxx	Gln	Gln	Pheol	**New**	
Lxx	Aib	Pro	Vxx	Aib	Aib	Gln	Gln	Pheol	**New** (trichosporin B IIIa – [Aib]^6^)	[64] [61]
									**New** (trichobrachin IIb A – [Ala]^6^)	[58] [59]
Lxx	Aib	Pro	Vxx	Aib	Aib	Gln	Gln	Pheol	**New** (suzukacillin A-11a – [Ala]^6^)	[60]
									**New** (trichosporin B-VIa – [Aib]^6^)	[61]
									**New** (trichosporin B-VIIa – [Aib]^6^)	[66]
Lxx	Aib	Pro	Vxx	Aib	Vxx	Gln	Gln	Pheol	**New** (trichosporin B-IVb – [Aib]^6^, trichosporin B-VIb – [Aib]^6^)	[61]
Lxx	Aib	Pro	Vxx	Aib	Vxx	Gln	Gln	Pheol	**New** (suzukacillin A-10b – [Ala]^6^)	[60]
Lxx	Aib	Pro	Vxx	Aib	Vxx	Glu	Gln	Pheol	**New**	
Lxx	Aib	Pro	Vxx	Aib	Vxx	Gln	Gln	Pheol	**New**	
Lxx	Aib	Pro	Vxx	Aib	Vxx	Glu	Gln	Pheol	**New**	
Lxx	Aib	Pro	Vxx	Aib	Vxx	Gln	Gln	Pheol	–	
Lxx	Aib	Pro	Vxx	Aib	Vxx	Gln	Gln	Pheol	–	
Lxx	Aib	Pro	Vxx	Aib	Vxx	Gln	Gln	Pheol	**New**	
Lxx	Aib	Pro	Vxx	Aib	Vxx	Gln	Gln	Pheol	**New**	

**Table 4:** Diagnostic Fragment Ions of 18- and 19-Residue Peptaibiotics Detected in the Ex-Type Culture (*CBS 119283*) of *Hypocrea phellinicola* (micrOTOF-Q II screening)

Diagnostic fragment ions	Peaks [*m/z*]a)							
	
	**11**	**12**	**13**	**14**	**15**	**16**	**17**	**18**

*t*_R_ [min]	30.9–31.1	31.8–32.0	32.2–32.6	32.5–32.7	32.8–33.1	35.1–35.3	37.0–37.2	37.7–37.9
[*M* + Na]^+^	1768.9850	1783.0115	1918.0846	1932.0976	1932.1017	1918.0877	1888.0616	1902.0891
[*M* + H]^+^	1747.0135	1761.0324	1896.0995	1910.1131	1910.1140	1896.1035	1866.0928	1880.1095
*a*_1_	100.0718	n.d.	n.d.	n.d.	n.d.	100.0720	100.0720	n.d.
*a*_3_	256.1647	256.1624	242.1508	256.1641	256.1707	242.1511	242.1506	256.1675
*a*_4_	n.d.	n.d.	n.d.	n.d.	327.1979	n.d.	n.d.	327.2046
*a*_5_	n.d.	n.d.	n.d.	n.d.	n.d.	n.d.	398.2312	n.d.
*b*_1_	128.0687	128.0658	128.0709	128.0708	128.0835	128.0715	128.0719	128.0721
*b*_2_	199.1075	199.1076	199.1109	199.1110	199.1161	199.1118	199.1115	199.1081
*b*_3_	284.1611	284.1617	270.1453	284.1622	284.1637	270.1434	270.1471	284.1634
*b*_4_	355.1972	355.1977	341.1846	371.1863	355.2023	357.1765	341.1815	355.1988
*b*_4_ – H_2_O	n.d.	n.d.	n.d.	353.1758	n.d.	339.1676	n.d.	n.d.
*b*_5_	426.2340	440.2546	426.2354	456.2441	440.2494	442.2277	426.2314	440.2546
*b*_6_	554.2840	568.3175	554.2840	584.3226	568.3175	570.2870	554.2989	568.3023
*b*_7_	639.3523	653.3691	639.3539	669.3625	653.3679	655.3443	639.3530	653.3685
*b*_8_	752.4400	766.4531	752.4386	782.4408	766.4563	768.4296	752.4353	766.4519
*b*_9_	837.4860	851.5024	837.4880	867.4961	851.5028	853.4825	837.4896	851.5066
*b*_10_	894.5048	908.5271	894.5061	924.5223	908.5250	910.5022	894.5076	908.5242
*b*_11_	1007.5856	1021.6063	1007.5967	1037.6039	1027.6073	1023.5862	1007.5917	1021.6085
*b*_12_	1092.6441	1106.6573	1092.6442	1122.6523	1106.6575	1108.6413	1092.6474	1106.6629
*b*_12_ – H_2_O	n.d.	n.d.	n.d.	n.d.	n.d.	1090.6265	1074.6077	1088.6332
*y*_6_	655.3841	655.3841	–	–	–	–	–	–
*y*_6_ – AA (18)	509.3130	509.3130	–	–	–	–	–	–
*y*_6_ – AA (18-17)	381.2540	381.2540	–	–	–	–	–	–
*y*_6_ – AA (18-16)	282.1709	282.1709	–	–	–	–	–	–
*y*_7_	–	–	804.4624	788.4706	804.4669	788.4697	774.4592	774.4593
*y*_7_ – H_2_O	–	–	786.4472	770.4510	n.d.	770.4510	756.4383	756.4383
*y*_7_ – AA (19)	–	–	637.3680	637.3708	637.3725	637.3705	623.3566	623.3559
*y*_7_ – AA (19-18)	–	–	509.3068	509.3140	509.3085	509.3103	495.2961	495.2962
*y*_7_ – AA (19-17)	–	–	381.2489	381.2515	381.2545	381.2513	367.2370	367.2373
*y*_7_ – AA (19-16)	–	–	n.d.	n.d.	282.1814	282.1814	282.1815	282.1815

**19**	**20**	**21**	**22**	**23**	**24**	**25**	**26**	**27**
38.3–38.4	38.8–39.2	39.8–40.1	40.6–40.9	41.5–41.6	42.1–42.3	43.4–43.6	44.2–44.4	45.0–45.6
902.0921	1916.1081	1917.1085	1930.1235	1931.1236	1944.1425	1958.1599	1958.1548	1972.1635
1880.1136	1894.1331	1895.1278	1908.1474	1909.1391	1922.1601	1936.1738	1936.1750	1950.1894
100.0721	100.0721	100.0747	100.0722	100.0722	100.0722	n.d.	n.d.	n.d.
242.1514	256.1682	256.1682	256.1677	256.1649	n.d.	n.d.	n.d.	n.d.
313.1832	327.2048	327.2049	327.2042	327.2050	n.d.	n.d.	n.d.	n.d.
n.d.	412.2533	412.2564	426.2817	n.d.	n.d.	n.d.	n.d.	n.d.
128.0722	128.0724	128.0718	128.0720	128.0708	128.0712	128.0672	128.0701	128.0684
199.1121	199.1081	199.1118	199.1083	199.1141	[255]	[269]	227.1404	241.1564
270.1476	284.1608	284.1608	284.1641	284.1631			312.1955	326.2055
341.1814	355.1988	355.1973	355.1972	355.1972	383.2306	397.2427	383.2297	397.2477
n.d.	n.d.	n.d.	n.d.	n.d.	n.d.	n.d.	n.d.	n.d.
426.2314	440.2531	440.2513	454.2685	454.2686	468.2836	482.3001	482.2988	496.3148
554.2989	568.3022	568.3119	582.3249	582.3249	596.3361	610.3531	610.3608	624.3766
639.3513	653.3673	653.3654	667.3841	667.3836	681.3976	695.4131	695.4110	709.4286
752.4346	766.4505	766.4489	780.4662	780.4659	794.4802	808.4949	808.4934	822.5109
837.4888	851.5044	851.5023	865.5205	865.5199	879.5335	893.5492	893.5457	907.5631
894.5075	908.5234	908.5216	922.5386	922.5395	936.5517	950.5713	950.5659	964.5813
1007.5920	1021.6065	1021.6039	1035.6228	1035.6231	1049.6347	1063.6526	1063.6516	1077.6661
1092.6463	1106.6606	1106.6578	1120.6786	1120.6785	1134.6898	1148.7069	1148.7051	1162.7188
1074.6284	1088.6331	1088.6424	1102.6441	1102.6440	1116.6595	1130.6997	1130.7031	1144.7051
–	–	–	–	–	–	–	–	–
–	–	–	–	–	–	–	–	–
–	–	–	–	–	–	–	–	–
–	–	–	–	–	–	–	–	–
788.4710	788.4710	789.4647	788.4718	789.4597	788.4710	788.4705	788.4678	788.4668
770.4509	770.4509	771.4475	770.4508	771.4390	770.4507	770.4507	770.1538	770.1538
637.3707	637.3707	638.3638	637.3705	638.3574	637.3721	637.3678	637.3649	637.3676
509.3096	509.3096	510.3014	509.3108	510.2964	509.3105	509.3113	509.3093	509.3082
381.2513	381.2513	381.2483	381.2524	381.2520	381.2505	381.2508	381.2506	381.2492
n.d.	n.d.	282.1837	282.1813	282.1813	282.1813	282.1920	282.1781	282.1917

a) n.d., Not detected.

#### 2.3.3. maXis *Screening*

All SF1 peptaibiotics, compounds **12, 14, 19, 28–47**, of the *ex-type* plate culture grown and analyzed at DTU *(Tables 5* and *6*, and *Fig. 2,c)* exhibit the characteristic deletion of the Ala/Aib residue in position 6. However, different positional isomers and homologues were found, *e.g*., the 17-residue deletion sequence **29**, lacking the C-terminal dipeptide [Gln^18^–Pheol^19^]. In compound **31**, a Ser-residue was found in position 3, whereas compound **30** exhibited a Gly residue in position 4. Overall, the structural diversity of peptaibiotics produced by the two cultures was much higher as compared to the specimen: variable amino acid residues were in positions 2,3, 4, 5, 6, 17, 18, and 20 *(Fig. 1,b)*.

**Table 5:** Sequences of 10-, 11-, 17-, 18-, and 19-Residue Peptaibiotics Detected in the *Ex-Type* Culture (*CBS 119283*) of *Hypocrea phellinicola* (maXis *screening*)

No.	*t*_R_ [min]	[*M* + *H*]^+^		Residue										
				
				1	2	3	4	5	6	7	8	9	10	11
**28**	10.8	1747.0131	Ac	Aib	Ala	Aib	Ala	Aib	–	Gln	Aib	Lxx	Aib	Gly
**12**	11.2	1761.0273	Ac	Aib	Ala	Aib	Ala	Aib	–	Gln	Aib	Lxx	Aib	Gly
**14**	12.2	1911.1213	Ac	Aib	Ala	Aib	Ser	Aib	–	Gln	Aib	Lxx	Aib	Gly
**29**	12.6	1632.9708	Ac	Aib	Ala	Aib	Ala	Aib	–	Gln	Aib	Lxx	Aib	Gly
**30**	13.0	1880.1000	Ac	Aib	Ala	Aib	Gly	Aib	–	Gln	Aib	Lxx	Aib	Gly
**31**	13.2	1882.0784	Ac	Aib	Ala	Ser	Ala	Aib	–	Gln	Aib	Lxx	Aib	Gly
**19**	13.5	1880.1008	Ac	Aib	Ala	Ala	Ala	Aib	–	Gln	Aib	Lxx	Aib	Gly
**32**	14.1	1896.0964	Ac	Aib	Ala	Ser	Ala	Aib	–	Gln	Aib	Lxx	Aib	Gly
**33**	14.9	1880.1035	Ac	Aib	Ala	Ala	Ala	Aib	–	Gln	Aib	Lxx	Aib	Gly
**34**	15.5	1866.0863	Ac	Aib	Ala	Ala	Ala	Aib	–	Gln	Aib	Lxx	Aib	Gly
**35**	15.9	1880.1012	Ac	Aib	Ala	Aib	Ala	Aib	–	Gln	Aib	Lxx	Aib	Gly
**36**	16.2	1867.0706	Ac	Aib	Ala	Ala	Ala	Aib	–	Gln	Aib	Lxx	Aib	Gly
**37**	16.4	1880.1007	Ac	Aib	Ala	Ala	Ala	Aib	–	Gln	Aib	Lxx	Aib	Gly
**38**	16.7	n.d.	Ac	Aib	Ala	Aib	Ala	Aib	–	Gln	Aib	Lxx	Aib	Gly
**39**	16.8	1880.1009	Ac	Aib	Ala	Aib	Ala	Aib	–	Gln	Aib	Lxx	Aib	Gly
**40**	17.0	n.d.	Ac	Aib	Ala	Aib	Ala	Aib	–	Gln	Aib	Lxx	Aib	Gly
**41**	17.2	1880.0997	Ac	Aib	Ala	Ala	Ala	Aib	–	Gln	Aib	Lxx	Aib	Gly
**42**	17.5	1894.1210	Ac	Aib	Ala	Aib	Ala	Vxx	–	Gln	Aib	Lxx	Aib	Gly
**43**	17.7	1895.1007	Ac	Aib	Ala	Aib	Ala	Aib	–	Gln	Aib	Lxx	Aib	Gly
**44**	18.0	1894.1177	Ac	Aib	Ala	Ala	Ala	Vxx	–	Gln	Aib	Lxx	Aib	Gly
**45**	18.6	1908.1341	Ac	Aib	Ala	Aib	Ala	Vxx	–	Gln	Aib	Lxx	Aib	Gly
**46**	20.0	1922.1467	[227]^a)^			Aib	Ala	Aib	–	Gln	Aib	Lxx	Aib	Gly
**47**	21.5	1936.1660	[241]^b)^			Aib	Ala	Aib	–	Gln	Aib	Lxx	Aib	Gly
**48**	22.0	1009.7031	Oc^c)^	Aib	Gly	Lxx	Aib	–	Gly	Lxx	Aib	Gly	Lxx	Lxxol
**49**	22.1–22.2	1066.7242	Oc	Aib	Gly	Lxx	Aib	Gly	Gly	Lxx	Aib	Gly	Lxx	Lxxol

									Compound identical or positionally isomeric with	Ref.
		
12	13	14	15	16	17	18	19	20		
Lxx	Aib	Pro	Vxx	Aib	Aib	Gln	Gln		**New** (trichocellin A-V–[Ala]^6^ – Pheol)	[67]
Lxx	Aib	Pro	Vxx	Aib	Vxx	Gln	Gln		**New** (trichocellin A-VI – [Ala]^6^ – Pheol)	[67]
Lxx	Aib	Pro	Vxx	Aib	Vxx	Gln	Gln	Pheol	**New**	
Lxx	Aib	Pro	Vxx	Aib	Vxx	Gln			**New** (**12** – [Gln]^18^)	
Lxx	Aib	Pro	Vxx	Aib	Vxx	Gln	Gln	Pheol	**New** (**17**: [Ala]^4^→[Gly]^4^)	
Lxx	Aib	Pro	Vxx	Aib	Aib	Gln	Gln	Pheol	**New**	
Lxx	Aib	Pro	Vxx	Aib	Vxx	Gln	Gln	Pheol	**New** (trichosporin B-IVb – [Aib]^6^, trichosporin B-VIb – [Aib]^6^)	[61]
Lxx	Aib	Pro	Vxx	Aib	Vxx	Gln	Gln	Pheol	**New**	
Lxx	Aib	Pro	Vxx	Aib	Vxx	Gln	Gln	Pheol	**New** (positional isomer of **19, 37**, and **41**)	
Lxx	Aib	Pro	Vxx	Aib	Aib	Gln	Gln	Pheol	**New** (positional isomer of **17**)	
Lxx	Aib	Pro	Vxx	Aib	Aib	Gln	Gln	Pheol	**New** (trichosporin B-VIa – [Aib]^6^, trichosporin B-VIIb – [Aib]^6^)	[61] [66]
Lxx	Aib	Pro	Vxx	Aib	Aib	Glu	Gln	Pheol	**New** (35: [Gln]^17^→[Glu]^17^)	
Lxx	Aib	Pro	Vxx	Aib	Vxx	Gln	Gln	Pheol	**New** (positional isomer of **19, 33**, and **41**)	
Lxx	Aib	Pro	Vxx	Aib	Aib	Glu	Gln	Pheol	**New** (**39**: [Gln]^17^→[Glu]^17^)	
Lxx	Aib	Pro	Vxx	Aib	Aib	Gln	Gln	Pheol	**New** (positional isomer of **35**)	
Lxx	Aib	Pro	Vxx	Aib	Vxx	Gln	Gln	Pheol	**New** (trichosporin B-VIIa – [Aib]^6^)	[66]
Lxx	Aib	Pro	Vxx	Aib	Vxx	Gln	Gln	Pheol	**New** (positional isomer of **19, 33**, and **37**)	
Lxx	Aib	Pro	Vxx	Aib	Aib	Gln	Gln	Pheol	**New**	
Lxx	Aib	Pro	Vxx	Aib	Vxx	Glu	Gln	Pheol	**New** (positional isomer of **40**)	
Lxx	Aib	Pro	Vxx	Aib	Vxx	Gln	Gln	Pheol	**New** (positional isomer of **45**)	
Lxx	Aib	Pro	Vxx	Aib	Vxx	Gln	Gln	Pheol	**New** (positional isomer of **44**)	
Lxx	Aib	Pro	Vxx	Aib	Vxx	Gln	Gln	Pheol	–	
Lxx	Aib	Pro	Vxx	Aib	Vxx	Gln	Gln	Pheol	–	
									**New**	
									Trichogin A IV	[68]
									Sequence 13 or 14 from *Trichoderma* cf. *strigosum* CBS 119777	[49]
									Partial sequence 4 from *Hypocrea citrina* CBS 853.70	[48]
									Partial sequence 4 from *Hypocrea vinosa* CBS 247.63	[48]

a) The N-terminal sequence of compound **46**, which is represented by a mass difference of 227 Da, could not be assigned.

b) The N-terminal sequence of compound **47**, which is represented by a mass difference of 241 Da, could not be assigned.

c) Oc, Tentatively assigned as *n*-octanoyl residue.

**Table 6:** Diagnostic Fragment Ions of 10-, 11-, 17-, 18-, and 19-Residue Peptaibiotics Detected in the *Ex-Type* Culture (*CBS 119283*) of *Hypocrea phellinicola* (maXis *screening*)

Diagnostic fragment ions	Peaks [*m/z*]a)					
	
	**28**	**12**	**14**	**28**	**30**	**31**

*t*_R_ [min]	10.8	11.2	12.2	12.6	13.0	13.2
[*M* + H]^+^	1747.0131	1761.0273	1911.1213	1632.9708	1880.1000	1882.0784
*b*_1_	n.d.	n.d.	n.d.	n.d.	n.d.	n.d.
*b*_2_	n.d.	199.1093	n.d.	199.1087	199.1087	199.1123
*b*_3_	284.1601	284.1607	284.1613	284.1609	284.1604	286.1389
*b*_4_	355.1989	355.1980	371.1938	355.1975	341.1819	357.1760
*b*_4_ – H_2_O	n.d.	n.d.	438.2353	n.d.	412.2541	424.2167
*b*_5_	440.2512	440.2509	456.2470	440.2506	426.2347	442.2296
*b*_6_	568.3097	568.3098	584.3039	568.3096	554.2926	570.2869
*b*_7_	653.3615	653.3626	669.3571	653.3619	639.3458	655.3404
*b*_8_	766.4456	766.4471	782.4415	766.4461	752.4294	768.4257
*b*_9_	851.5003	851.4996	867.4953	851.4987	837.4826	853.4789
*b*_10_	908.5192	908.5208	924.5190	908.5199	894.5026	910.4971
*b*_11_	1021.6077	1021.6046	1038.5981	1021.6053	1007.5901	1023.5860
*b*_12_	1106.6578	1106.6578	1122.6537	1106.6590	1092.6412	1108.6356
*b*_12_ – H_2_O	n.d.	n.d.	n.d.	n.d.	n.d.	n.d.
*y*_5_	–	–	–	527.3191	–	–
*y*_5_ – AA (17)	–	–	–	381.2497	–	–
*y*_5_ – AA (17-16)	–	–	–	282.1814	–	–
*y*_5_ – AA (17-15)	–	–	–	197.1287	–	–
*y*_6_	641.3626	655.3768	–	–	–	–
*y*_6_ – AA (18)	495.2923	509.3095	–	–	–	–
*y*_6_ – AA (18-17)	367.2353	381.2500	–	–	–	–
*y*_6_ – AA (18-16)	282.1812	282.1816	–	–	–	–
*y*_6_ – AA (18-15)	197.1274	197.1288	–	–	–	–
*y*_7_	–	–	788.4676	–	788.4676	774.4501
*y*_7_ – H_2_O	–	–	637.3673	–	637.3673	623.3515
*y*_7_ – AA (19)	–	–	509.3117	–	509.3117	495.2926
*y*_7_ – AA (19-18)	–	–	381.2509	–	381.2509	367.2344
*y*_7_ – AA (19-17)	–	–	282.1814	–	282.1814	282.1813
*y*_7_ – AA (19-16)	–	–	197.1284	–	197.1284	197.1270

**19**	**32**	**33**	**34**	**35**	**36**

13.5	14.1	14.9	15.5	15.9	16.2
1880.1008	1896.0964	1880.1035	1866.0863	1880.1012	1867.0706
n.d.	n.d.	n.d.	128.0697	n.d.	n.d.
199.1123	199.1123	199.1123	199.1074	199.1078	199.1078
270.1449	286.1389	270.1449	270.1449	284.1605	270.1438
341.1826	357.1760	341.1826	341.1819	355.1975	341.1816
n.d.	424.2191	408.2242	408.2280	422.2402	n.d.
426.2349	442.2296	426.2349	426.2349	440.2506	426.2354
554.2934	570.2869	554.2934	554.2933	568.3087	554.2932
639.3463	655.3404	639.3463	639.3465	653.3621	639.3461
752.4301	768.4257	752.4301	752.4303	766.4461	752.4295
837.4813	853.4789	837.4813	837.4833	851.4992	837.4824
894.5075	910.4971	894.5075	894.5044	908.5203	894.5037
1007.5825	1023.5860	1007.5825	1007.5891	1021.6041	1007.5911
1092.6420	1108.6370	1092.6440	1092.6432	1106.6582	1092.6413
n.d.	n.d.	n.d.	n.d.	n.d.	n.d.
–	–	–	–	–	–
–	–	–	–	–	–
–	–	–	–	–	–
–	–	–	–	–	–
–	–	–	–	–	–
–	–	–	–	–	–
–	–	–	–	–	–
–	–	–	–	–	–
–	–	–	–	–	–
788.4661	788.4667	788.4668	774.4504	774.4503	775.4366
637.3669	637.3683	637.3683	623.3499	623.3499	624.3356
509.3092	509.3078	509.3078	495.2931	495.2931	496.2769
381.2498	381.2500	381.2500	367.2337	367.2337	367.2337
282.1806	282.1815	282.1815	282.1812	282.1812	282.1814
197.1288	197.1286	197.1286	197.1284	197.1284	197.1287

Diagnostic fragment ions	Peaks [*m/z*]a)					
	
	**37**	**38**	**39**	**40**	**41**	**42**

*t*_R_ [min]	16.4	16.7	16.8	17.0	17.2	17.5
[*M* + *H*]^+^	1880.1007	n.d.	1880.1009	n.d.	1880.0997	1894.1210
*b*_1_	n.d.	n.d.	128.0701	128.0713	n.d.	n.d.
*b*_2_	199.1075	199.1075	199.1077	199.1075	199.1075	199.1080
*b*_3_	270.1444	284.1603	284.1602	284.1599	270.1444	284.1604
*b*_4_	341.1819	355.1974	355.1975	355.1973	341.1819	355.1974
*b*_4_ – H_2_O	n.d.	n.d.	422.2399	n.d.	n.d.	436.2493
*b*_5_	426.2350	440.2504	440.2499	440.2504	426.2350	454.2659
*b*_6_	554.2935	568.3091	568.3080	568.3086	554.2935	582.3240
*b*_7_	639.3462	653.3619	653.3613	653.3615	639.3462	667.3770
*b*_8_	752.4307	766.4459	766.4450	766.4452	752.4307	780.4612
*b*_9_	837.4843	851.4983	851.4983	851.4987	837.4843	865.5140
*b*_10_	894.5019	908.5197	908.5205	908.5230	894.5019	922.5363
*b*_11_	1007.5901	1021.6066	1021.6041	1021.6054	1007.5901	1035.6190
*b*_12_	1092.6420	1106.6569	1106.6577	1106.6577	1092.6420	1120.6761
*b*_12_ – H_2_O	n.d.	n.d.	n.d.	1088.6517	n.d.	1103.6621
*y*_5_	–	–	–	–	–	–
*y*_5_ – AA (17)	–	–	–	–	–	–
*y*_5_ – AA (17-16)	–	–	–	–	–	–
*y*_5_ – AA (17-15)	–	–	–	–	–	–
*y*_6_	–	–	–	–	–	–
*y*_6_ – AA (18)	–	–	–	–	–	–
*y*_6_ – AA (18-17)	–	–	–	–	–	–
*y*_6_ – AA (18-16)	–	–	–	–	–	–
*y*_6_ – AA (18-15)	–	–	–	–	–	–
*y*_7_	788.4660	775.4348	774.4505	788.4664	788.4664	774.4522
*y*_7_ – H_2_O	637.3704	624.3348	623.3515	637.3670	637.3670	623.3499
*y*_7_ – AA (19)	509.3084	469.2766	495.2929	509.3079	509.3079	495.2931
*y*_7_ – AA (19-18)	381.2504	367.2338	367.2338	381.2493	381.2493	367.2337
*y*_7_ – AA (19-17)	282.1806	282.1808	282.1808	282.1807	282.1807	282.1812
*y*_7_ – AA (19-16)	197.1288	197.1283	197.1274	197.1282	197.1282	197.1284

**43**	**44**	**45**	**46**	**47**	**48**	**49**

17.7	18.0	18.6	20.0	21.5	22.0	22.1–22.2
1895.1007	1894.1177	1908.1341	1922.1467	1936.1660	1009.7031	1066.7242
n.d.	128.0684	128.0684	n.d.	n.d.	n.d.	n.d.
199.1084	199.1074	199.1080	227.1386	241.1536	212.1663	212.1644
284.1606	270.1440	284.1604	312.1916	326.2076	269.1858	269.1850
355.1969	341.1818	355.1974	383.2288	397.2443	382.2698	382.2695
n.d.	422.2401	436.2550	n.d.	n.d.	–	–
440.2499	440.2501	454.2659	468.2807	482.2975	467.3234	467.3230
568.3077	568.3087	582.3240	596.3410	610.3540	524.3442	524.3428
653.3609	653.3614	667.3770	681.3925	695.4084	637.4289	581.3654
766.4466	766.4453	780.4612	794.4774	808.4926	722.4814	694.4498
851.4985	851.4983	865.5140	879.5284	893.5450	779.5027	779.5029
908.5184	908.5202	922.5363	936.5518	950.5672	892.5860	836.5243
1021.6039	1021.6067	1035.6190	1049.6372	1063.6524	–	949.6064
1106.6577	1106.6590	1120.6744	1134.6878	1148.7083	–	–
1088.6389	n.d.	1102.6586	n.d.	n.d.	–	–
–	–	–	–	–	–	–
–	–	–	–	–	–	–
–	–	–	–	–	–	–
–	–	–	–	–	–	–
–	–	–	–	–	–	–
–	–	–	–	–	–	–
–	–	–	–	–	–	–
–	–	–	–	–	–	–
–	–	–	–	–	–	–
789.4503	788.4660	788.4670	788.4660	788.4650	–	–
638.3516	637.3677	637.3670	637.3677	637.3678	–	–
510.2927	509.3076	509.3079	509.3076	509.3077	–	–
381.2498	381.2495	381.2493	381.2495	381.2492	–	–
282.1814	282.1807	282.1807	282.1807	282.1814	–	–
197.1292	197.1284	197.1282	197.1284	197.1277	–	–

a) n.d., Not detected.

### 2.4. Lipopeptaibols as Trace Components in the Plate Cultures

Two lipopeptaibols, compounds **48** and **49**, were produced as trace components in the DTU plate culture. Compound **49** probably represents trichogin A IV [68] [69] or a positional isomer thereof. The new positionally isomeric compound **48**, named ‘lipophellin 1’, is characterized by the deletion of [Gly]^5^ of compound **49** *(Tables 5* and *6*, and *Fig. 2,c)*.

## 3. Discussion

### 3.1. Hypophellins, Novel Long-Chain Peptaibiotics from *T. phellinicola*

The most notable result of this investigation is, indeed, the unequivocal confirmation of peptaibiotic biosynthesis in the natural habitat of *T phellinicola* growing on its host *Phellinus ferruginosus*, commonly known as the Rusty Porecrust. We here describe for the first time the *in vivo* detection of non-ribosomal peptide antibiotics5), which may significantly contribute to the complex interaction of a fungicolous ascomycete growing on its basidiomycetous host.

### 3.2. The Peptaibiome of the Specimen

The teleomorph produced a microheteroge-neous mixture of ten 20-residue HPHs, four of which, **6, 8, 9**, and **10**, are new (Table 1). Compared to smaller sequences consisting of less than 17 residues, long-chain peptaibiotics display a higher membrane-pore-formation activity by several orders of magnitude [71].

Depending on the individual sequence, seven to nine Aib residues are present, which strongly promote the formation of helical structures. i.e., α- or *3*_10_-helices, and even mixed forms [72–74], which is due to the steric constraints imposed by the geminal Me groups of the C^α^-atom [75]. All of them exhibit the structurally important features, which are required for the formation of transmembrane ion channels in artificial lipid bilayer membranes, as compiled by *Duclohier* [76], and *Duclohier* and *Wróblewski* [77]. A multitude of bioactivities has been described for 20-residue peptaibols of similar structure, which are compiled in *Table 7*.

**Table 7:** Biological Activities of Selected 20-Residue Peptaibols Structurally Closely Related to Hypophellins

Peptaibols	Bioactivities reported	Ref.
Longibrachins	Ion-channel formation in BLM, antimycoplasmic	[53]

Suzukacillins	Antibacterial, antifungal	[78]
	Ion-channel formation in BLM	[79]
	Haemolysis of human erythrocytes	[80]

Trichoaureocins	Haemolysis of sheep erythrocytes, antibacterial (g^+^)	[54]
Trichobrachins	Antibacterial (g^+^ )	[57]
Trichocellins	Induction of Ca^2+^-dependent catecholamine secretion from bovine adrenal medullary chromaffin cells	[67]
	Ion-channel formation in BLM	[81]

Trichokonins	Agonist towards Ca^2+^-channels in bullfrog cardiac myocytes	[55] [82]
	Antibacterial (g^+^ ), antifungal	[83]
	Induction of defense responses and systemic resistance in tobacco against tobacco mosaic virus	[46]
	Induction of apoptotic programmed cell death in fungal plant pathogens	[47]

Trichosporins B	Uncoupling of the respiratory activity of rat liver mitochondria	[64] [84]
	Induction of Ca^2+^-dependent catecholamine secretion from bovine adrenal medullary chromaffin cells	[85–87]
	Ion-channel formation in BLM	[88]
	Antitrypanosomal	[66]

Paracelsins	Antibacterial (g^+^ )	[89]
	Increasing digestibility of starch and cellulose in ruminants; haemolysis of human erythrocytes; acutely toxic in mice (*LD*_50_ 5 mg/kg, *i.p*.)	[90]
	Mosquitocidal (larvae of *Culex pipiens*)	[91]
	Toxic against aquatic invertebrates (*Daphnia magna, Artemisia salina*)	[92] [93]
	Ion-channel formation in BLM	[71]
	Antifungal	[93]

### 3.3. The Peptaibiome of the *Ex-Type* Plate Culture

In contrast to what has been observed for the specimen, 20-residue peptaibols could not be detected. Instead, fifteen 19-residue peptaibols were detected in the *micrOTOF-Q II* screening and another eighteen in the *maXis* screening. Although sequences of **11–47** still exhibit the characteristic building scheme of SF1, they are distinguished from the 20-residue peptaibols of the teleomorph specimen by a deletion of the Aib/Ala residue in position 6 (Δ Ala/Aib6) of the peptide chain. This deletion, however, is predicted not to negatively influence the bioactivity of these long-chain peptaibols, as all important structural features are still present, which comply with the requirements for the formation of transmembrane ion channels in artificial lipid bilayer membranes [76] [77]. The three 18-residue sequences, **11, 12,** and **28**, exhibit a deletion of the C-terminal amino alcohol, whereas the dipeptide [Gln^18^–Pheol^19^] is deleted in **29**, a 17-residue sequence. Truncated versions of SF1 peptaibols lacking the C-terminal amino alcohol or even the adjacent Gln residue have been reported before.

The ten 19-residue peptaibiotics, trichobrachins I (TB I), lacking the C-terminal Pheol residue, as well as the two 18-residue trichobrachins II-1 and -2 (TB II), which exhibit a deletion of the C-terminal dipeptide [Gln^19^–Pheol^20^], were shown to originate from 20-residue trichobrachins II (TB II) by enzymatic degradation [57]. Two minor desPheol compounds F30, representing 1.3% of the alamethicin (ALM) mixture investigated, have been detected by non-aqueous capillary electrophoresis (NACE) coupled to electrospray mass spectrometry [94].

### 3.4. l-Phenylalaninol as Constituent of Natural Products

C-Terminal l-Pheol is commonly found in peptaibiotics [17] [18] but has also been infrequently reported as a constituent of other plant and fungal secondary metabolites such as *N*-benzoyl-l-phenylalaninol from *Catharanthus pusillus* [95] and *Diospyros quaesita* [96], *O*-acetyl-*N*-(*N*′-benzoyl-l-phenylalanyl)-l-phenylalaninol from *Euphorbia fischeriana* and *E. kansui* [97], and *N*-benzoyl-*O*-[*N*′-benzoyl-l-phenylalanyl]-l-phenylalaninol from *Penicillium arenicola* (syn. *P. canadense)* [98].

### 3.5. l-Tyrosinol as a Constituent of Natural Products

To the best of our knowledge, neither d- nor l-tyrosinol^6^) has ever been reported as constituent of either linear or cyclic peptides of microbial origin, including peptaibiotics. However, l-tyrosinol is a ‘cryptic’ building block of the following natural products:

- farinosone C, an amide from *Paecilomyces farinosus* RCEF 0101 [99];
- cordyceamides A and B from a liquid culture of *Cordyceps sinensis* [100];
- preoxazinin-7, the linear precursor [101], and cyclic oxazinins from the digestive glands of *Mytilus galioprovincialis* [102] [103].

### 3.6. The Lifestyle of *Trichoderma phellinicola*: Findings and Thoughts

Taken these findings together, we dare predict a mycoparasitic lifestyle of the host-specific polyporicolous *Trichoderma phellinicola:*

It has been demonstrated by *in vitro* studies that chitinases and β-1,3-glucanases act synergistically with peptaibiotics in inhibiting spore germination and hyphal elongation of *Botrytis cinerea*. Parallel formation of hydrolytic enzymes and 19-residue antifungal trichorzianins A and B by the potent mycoparasite *Trichoderma atroviride*7) is triggered in the presence of cell walls of plant-pathogenic fungi [106]. Trichorzianins have previously been shown to form voltage-gated ion channels in planar lipid bilayers [107] and to modify the membrane permeability of liposomes, and they are active against *Rhizoctonia solani* and *Phythophthora cactorum* [108]. Based on these findings, a model of how peptaibiotics such as trichorzianins and hydrolases interact synergistically was proposed.

First, the host cell wall is digested enzymatically; thereafter, peptaibiotics will penetrate the cell membrane to form ion channels. Cell leakage reduces the ability of the host to effectively repair its cell wall. Eventually, inhibition of chitin and β-glucan synthesis further amplifies the destructive effect of chitinases and β-1,3-glucanases [108]. These mechanisms, however, may also account for the recently published induction of programmed cell death in plant fungal pathogens [47] caused by the 20-residue peptaibol trichokonin VI (= gliodeliquescin A [56])8), from *T. koningii, T. pseudokoningii*, and *T. deliquescens* (syn. *Gliocladium deliquescens*) [20]. The presence of peptaibiotics was also shown to play a role in the induction of plant defence responses [110].

### 3.7 Remarks on Non-Ribosomal Biosynthesis and Module Skipping by *T. phellinicola*

The exclusive production of 20-residue peptaibols by the *T. phellinicola* teleomorph indicates the presence of a 20-module NRPS. As the culture CBS 119283 has been shown to produce 17-, 18-, and 19-residue peptaibiotics only, it is likely to contain a 19-module NRPS, lacking the 6th module activating Ala or Aib. In addition, modules 3 and 4 show differing substrate specificities, as compared to the teleomorph, thus permitting the incorporation of Ala or Ser in position 3 and of Gly, Ala, or Ser in position 4, respectively. These findings indicate substantial variations in the sequences of the SF1-type peptaibol synthetases of both strains. As has been discussed in the case of SF4-type peptaibols, genes involved in secondary-metabolite products show a much broader sequential variety than housekeeping genes [50]. We here, indeed, find evidence for a significant structural variation within a large gene.

**Experimental Part**

*Chemicals*. All solvents used, MeCN (99.9%), MeOH (99.9%), CH_2_Cl_2_ (99.8%), and HCOOH (98%), were of LC/MS grade from *Sigma-Aldrich* (D-Steinheim). Water was purified by a *Merck-Millipore Milli-Q Synthesis A10* system (D-Schwalbach/Ts.).

*Origin of Specimen*. The teleomorphic specimen of *Trichoderma phellinicola* growing on its host *Phellinus ferruginosus* was collected in the ‘Národni park Podyjí’ (Czech Republic, Moravia), near Hardegg at the bridge across the River Thaya, just across the border between Austria and the Czech Republic.

*Origin of* Trichoderma phellinicola *CBS 119283* (ex-type). All details concerning this new species were given by *Jaklitsch* [20].

*Extraction of Specimens*. The teleomorph was extracted with CH_2_Cl_2_/MeOH 1:1 (*v/v*), the solvent was evaporated *in vacuo (Rotavapor R-215, Biichi*, D-Essen), and the extract was cleaned up over *Sep-Pak Classic C_18_* cartridges *(Waters*, D-Eschborn) as described by *Krause et al*. [48].

*Cultivation and Extraction of Pure Cultures*. Cultures of the specimen were grown on potato dextrose agar (PDA; *Becton Dickinson*, D-Heidelberg) at 23° for 6 d. These subcultures were used for inoculation of the main cultures. After 10 d of cultivation at 23° in the dark, main cultures were extracted as described for the teleomorph.

*LC/MS Analysis*. Two QTOF systems, both from *Bruker Daltonic* (D-Bremen) controlled by HyStar v. 3.2 were used. Both instruments were equipped with an orthogonal ESI source and coupled to a *Dionex UltiMate 3000* UHPLC (*Dionex*, D-Idstein).

*System 1:* high-resolution *micrOTOF Q-II* mass spectrometer. For separation, an *Acclaim* 120 *C_8_*, 3 μm, 2.1 × 150 mm, column (*Dionex*, D-Idstein) at a flow rate of 0.25 ml/min^−1^ and a temp. of 35° was used. Eluent *A* consisted of H_2_O + 0.1% HCOOH and eluent *B* of 95% MeCN + 0.1% HCOOH. Subsamples of 10 μl were injected. The column was held at 80% *A*/20% *B* for 5 min, then a gradient from 20% *B* to 100% over 55 min was applied. Thereafter, the column was held at 100% *B* for 15 min, returned to the start conditions in 1 min, and finally equilibrated for 14 min.

Samples were screened for peptaibiotics in the positive-ion mode using the following three-step routine procedure: first a full scan was recorded from *m/z* 50 to 3000. In *System 1*, this was followed by CID measurements from *m/z* 50 to 2000, recorded at energy of 150 eV. Finally, results of CID-MS were verified by MS/MS experiments on selected precursor ions. For precursors of *m/z* < 1000, a collision energy of 30 eV was applied, precursor ions in the *m/z* range from 1000 to 1500 were fragmented at a collision energy of 35 eV and precursor ions of *m/z* > 1500 at a collision energy of 40 eV. The isolation width for MS/MS experiments was set to ± 1 Da.

*System 2:* The *maXis 3G QTOF* mass spectrometer operated at a resolution of 40,000 FWHM. An *Acquity* BEH300 *C_18_*,1.7 μm, 2.1 × 150 mm, column (*Waters*, D-Eschborn) was used for separation, using H_2_O + 0.1% HCOOH (eluent *A*) and 100% MeCN + 0.1% HCOOH(eluent *B*). The flow rate was set to 0.3 ml/min and the temp. to 40°. The gradient started with 90% *A*/10% *B* and was changed to 50% *A*/50% *B* at 7 min, then to 30% *A*/70 % *B* at 25 min, then raised to 100% *B* at 38 min, and held at 100% *B* until 41 min before setting to starting conditions from time 42 min to 46 min. Three μl were injected. MS were scanned in the *m/z* range of 100–2,000. Auto MS with precursor ion-dependent collision energy optimization was used for fragmentation in the range of 10–65 eV.

Data interpretation was performed using the DataAnalysis v. 4.0 software (*Bruker Daltonic*, D-Bremen). Use of high-resolution (HR)ESI-MS allowed the unequivocal sequencing of fragment-ion series according to the *Roepstorff/Fohlman–Biemann* nomenclature. In cases where the isomeric amino acids (Leu/Ile and Val/Iva, resp.) or the corresponding amino alcohols (Leuol/Ileol) with the same elemental composition could not be distinguished, the abbreviations Lxx, Vxx, and Lxxol were used instead [48–50].

This study was supported by the Hessian Ministry for Science and Art by a grant from the *LOEWE-*Schwerpunkt program ‘*Insect Biotechnology*’ to *A. V*. DTU acknowledges the grant from the *Danish Research Council* (FI 2136-08-0023) for the *maXis* QTOF system, and MYCORED (EC KBBE-2007-222690-2) for supporting *A. I*. Support by the *Austrian Science Fund* (FWF; project P22081-B17) is acknowledged by *W. M. J*. The authors are indebted to Prof. Dr. *Hartmut Laatsch* (Institute of Organic and Biomolecular Chemistry, University of Göttingen, Germany) for his valuable comments on the occurrence of tyrosinol as a constituent of natural products.

## Footnotes

4) These subfamilies were introduced at a time when the total number of peptaibiotics described did not exceed 200 sequences. As of October 2012, *ca*. 1,000 individual sequences are known, which also exhibit new building schemes and constituents. Consequently, there is an urgent need to reconsider this classification.

5) Hypophellins were simultaneously detected in an LC/MS/MS screening of 15 specimens belonging to nine *Hypocrea* species, which have been collected in their natural habitat. Recently, a manuscript on the *in vivo* detection of hypopulvins, novel peptaibiotics from the polyporicolous fungus *H. pulvinata*, has been published. The results therein corroborate that peptaibiotics are produced by a fungicolous fungus during infection of its natural hosts [70].

6) C-Terminal β-amino alcohols with the d-configuration have not yet been reported for peptaibiotics.

7) The trichorzianin-producing strain ATCC 36042 (= CBS 391.92) was originally identified as *T. harzianum* [104] but later shown to belong to *T. atroviride* [105]. The high degree of misidentification of *Trichoderma* species prior to introduction of phylogenetic analysis is still regarded a major problem, unless authors describe how their cultures were identified [17].

8) Gliodeliquescin A has been isolated from *Gliocladium deliquescens* NRRL 1086 [109] and not from NRRL 3091 [56]. According to phylogenetic data (18S-rRNA, and ITS 1 and 2), *G. deliquescens* NRRL 1086 (= CBS 228.48 = ATCC 10097) was re-identified as *G. viride* (http://www.straininfo.net/strains/260309).
